# Endogenous annexin A1 is a novel protective determinant in nonalcoholic steatohepatitis in mice

**DOI:** 10.1002/hep.27141

**Published:** 2014-05-12

**Authors:** Irene Locatelli, Salvatore Sutti, Aastha Jindal, Marco Vacchiano, Cristina Bozzola, Chris Reutelingsperger, Dennis Kusters, Stefania Bena, Maurizio Parola, Claudia Paternostro, Elisabetta Bugianesi, Simon McArthur, Emanuele Albano, Mauro Perretti

**Affiliations:** 1 Department of Health Sciences and Interdisciplinary Research Center for Autoimmune Diseases, University “Amedeo Avogadro” of East PiedmontNovara, Italy; 2 Cardiovascular Research Institute MaastrichtDepartment of Biochemistry, Maastricht UniversityMaastricht, The Netherlands; 3 William Harvey Research Institute, Queen Mary University of London, London, United Kingdom; and Departments of TurinItaly; 4 Clinical and Biological Sciences, University of TurinTurin, Italy; 5 Medical Sciences, University of TurinTurin, Italy

## Abstract

Annexin A1 (AnxA1) is an effector of the resolution of inflammation and is highly effective in terminating acute inflammatory responses. However, its role in chronic settings is less investigated. Because changes in AnxA1 expression within adipose tissue characterize obesity in mice and humans, we queried a possible role for AnxA1 in the pathogenesis of nonalcoholic steatohepatitis (NASH), a disease commonly associated with obesity. NASH was induced in wild-type (WT) and AnxA1 knockout (AnxA1 KO) C57BL/6 mice by feeding a methionine-choline deficient (MCD) diet up to 8 weeks. In MCD-fed WT mice, hepatic AnxA1 increased in parallel with progression of liver injury. This mediator was also detected in liver biopsies from patients with NASH and its degree of expression inversely correlated with the extent of fibrosis. In both humans and rodents, AnxA1 production was selectively localized in liver macrophages. NASH in AnxA1 KO mice was characterized by enhanced lobular inflammation resulting from increased macrophage recruitment and exacerbation of the M1 phenotype. Consistently, *in vitro* addition of recombinant AnxA1 to macrophages isolated from NASH livers down-modulated M1 polarization through stimulation of interleukin-10 production. Furthermore, the degree of hepatic fibrosis was enhanced in MCD-fed AnxA1 KO mice, an effect associated with augmented liver production of the profibrotic lectin, galectin-3. Accordingly, AnxA1 addition to isolated hepatic macrophages reduced galectin-3 expression. *Conclusions*: Macrophage-derived AnxA1 plays a functional role in modulating hepatic inflammation and fibrogenesis during NASH progression, suggesting the possible use of AnxA1 analogs for therapeutic control of this disease.

Nonalcoholic fatty liver disease (NAFLD) is regarded as the hepatic feature of so-called metabolic syndrome (MetS) and is becoming the most common form of liver injury worldwide as a result of the diffusion of overweight and obesity.^1^ In approximately 10%-20% of patients with NAFLD, the disease evolves with the development of hepatocellular damage and lobular inflammation, often leading to hepatic fibrosis and cirrhosis.^1^ Available evidence indicates that the mechanisms responsible for the evolution of NAFLD to nonalcoholic steatohepatitis (NASH) may involve lipotoxicity caused by increased circulating free fatty acids, oxidative damage, and endoplasmic reticulum stress.^2^,^3^ These factors not only cause hepatocyte death, but can also stimulate Kupffer cells to secrete inflammatory mediators that, in turn, recruit and activate leucocytes within the liver.^2^,^3^ Nonetheless, the mechanisms responsible for persistence of hepatic inflammation along with those leading to the evolution of NASH to fibrosis are still incompletely characterized. It is increasingly evident that a failure in the mechanisms responsible for terminating inflammatory responses might result in chronic inflammation.^4^

Resolution of an acute inflammation is orchestrated by a variety of protein and autacoids that down-modulate leukocyte recruitment, promote clearance of tissue leucocytes, and switch macrophage phenotype favoring tissue healing.^5^ Among these proresolving factors, Annexin A1 (AnxA1), also known as lipocortin-1, is receiving increasing attention. AnxA1 is a 37-kDa calcium-phospholipid–binding protein highly expressed in myeloid cells and regulated by glucocorticoids.^6^ By interacting with its receptor, formyl peptide receptor 2/lipoxin A_4_ receptor (FPR2/ALX), AnxA1 down-regulates the production of proinflammatory mediators, such as eicosanoids, nitric oxide, and interleukin (IL)-6, reduces neutrophil migration to inflammatory sites, and promotes the clearance of apoptotic granulocytes.^6^,^7^ Furthermore, recent works suggest that endogenous AnxA1 may orchestrate epithelial repair^8^,^9^ and even counteract the development of lung fibrosis.^10^

Interest for a possible involvement of AnxA1 in the evolution of NAFLD originates from the observation that plasma AnxA1 levels are decreased in obese subjects inversely correlating with body mass index as well as with the inflammation marker, C-reactive protein,^11^ whereas an increased AnxA1 expression was observed in adipose tissue of obese mice.^12^ Furthermore, AnxA1 deficiency promotes adiposity and insulin resistance (IR) in Balb/c mice on a high-fat diet (HFD).^12^ Because AnxA1-null mice also display inappropriate experimental inflammatory responses,^7^ we have investigated the possible role of AnxA1 in the evolution of experimental NASH induced by feeding mice with a methionine-choline deficient (MCD) diet.

## Material and Methods

### Animal and Experimental Protocol

Eight-week-old male AnxA1 knockout (AnxA1 KO) mice on a C57BL/6 background and wild-type (WT) animals were purchased from Charles River Laboratories (Charles River UK Ltd., Margate, UK) and fed for 4 or 8 weeks with either MCD or control diets (Laboratorio Dottori Piccioni, Gessate, Italy). In some experiments, mice also received a high-fat (35% w/v) liquid diet for 12 weeks (see Supporting Information for details). Experiments were approved by the Italian Ministry of Health and by the university commission for animal care following the criteria of the Italian National Research Council.

### Human Specimen Collection and Analysis

Liver biopsies from 28 consecutive patients with NAFLD or NASH referring to the Division of Gastro-Hepatology of the University of Turin (Turin, Italy) in the period April–November 2011 were analyzed. Specimens were collected at the time of first diagnosis and processed for histophatology and extraction of nucleic acid. Patients were characterized by anthropometric, clinical, and biochemical data, and liver biopsies were evaluated for severity of steatohepatitis fibrosis.^13^ All subjects gave informed consent to the analysis and the study was planned according to the guidelines of the local ethical committee. The major clinical and biochemical parameters are reported in Supporting Table 1.

### Biochemical Analysis

Plasma alanine aminotransferase (ALT) and liver triglycerides (TGs) were determined by spectrometric kits supplied by Radim S.p.A. (Pomezia, Italy) and Sigma Diagnostics (Milano, Italy), respectively. Circulating tumor necrosis factor alpha (TNF-α) and liver IL-12 levels were evaluated by commercial enzyme-linked immunosorbent assay (ELISA) kits supplied by Peprotech (Milano, Italy) and R&D Systems (Abingdon, UK), respectively.

### Histology and Immunohistochemistry

Lobular inflammation was scored blind, according to Kleiner et al.,^13^ in hematoxilin and eosin (H&E)-stained sections. Necroinflammatory foci and apoptotic cells were counted as reported previously.^14^ Liver macrophages and activated hepatic stellate cells (HSCs) were evidenced in formalin-fixed sections using, respectively, anti-mouse F4/80 or anti-human CD68 (eBioscience, San Diego CA) and α-smooth muscle actin (α-SMA) polyclonal antibodies (Abs; Labvision; Bio-Optica SpA, Milan, Italy) in combination with peroxidase-linked goat anti-rat immunoglobulin G (IgG) or horseradish peroxidase polymer kit (Biocare Medical, Concord, CA). AnxA1, FPR2/ALX, and galectin-3 were detected using specific Abs from Zymed Laboratories-Invitrogen (Carlsbad, CA), Santa Cruz Biotechnology (Dallas, TX), and R&D Systems (Minneapolis, MN), respectively.

### Real-Time Polymerase Chain Reaction Analyses

Liver RNA was retrotranscripted with a High Capacity complementary DNA (cDNA) Reverse Transcription Kit (Applied Biosystems Italia, Monza, Italy). Real-time polymerase chain reaction (RT-PCR) was performed in a Techne TC-312 termalcycler (TecneInc, Burlington, NJ) using TaqMan Gene Expression Master Mix and TaqMan Gene Expression probes for mouse TNF-α, IL-12p40, IL-23p19, IL-10, chemokine (C-C motif) ligand 2 (CCL2), C-C chemokine receptor type 2 (CCR2), inducible nitric oxide synthase (iNOS), arginase-1, macrophage galactose *N*-acetyl-galactosamine-specific lectin-1 (MGL-1), AnxA1, Fpr2/3 (orthologs of human FPR2/ALX),^14^ galectin-3, α1-procollagen, transforming growth factor beta 1 (TGF-β1), α-SMA, tissue inhibitor of metalloproteinase-1 (TIMP-1), matrix metalloproteinase (MMP)-9, MMP-13, and β-actin (Applied Biosystems Italia, Monza, Italy). All samples were run in duplicate, and the relative gene expression calculated as 2^−ΔCt^ is expressed as fold increase over control samples. Human sample analysis was performed using SsoFast EvaGreen Supermix (Bio-Rad, Hercules, CA), following the manufacturer's instructions. The sequence of primers used was: sense, 5′-GCAGGCCTGGTTTATTGAAA-3′; reverse, 5′-GCTGTGCATTGTTTCGCTTA-3′. Values were normalized to those of β-actin and are expressed by using the comparative 2^ΔΔCt^ method.

### AnxA1 Recombinant Protein Purification

cDNA of human AnxA1 carrying a cleavable *N*-terminal poly-His tag was expressed in *Escherichia coli* and purified as previously reported.^9^ Purity of recombinant AnxA1, as assesed by sodium dodecyl sulfate polyacrylamide gel electrophoresis and matrix-assisted laser desorption/ionization dual time-of-flight mass spectrometry, was >95%.

### Isolation and Purification of Liver Macrophages

Macrophages were purified from livers of either controls or 4-week MCD-fed mice, as previously described.^15^ Cell purity was above 80%, as determined by flow cytometry (FCM), after immunostaining for CD45 and F4/80. Cells were incubated overnight with AnxA1 (100 nM) in the presence or absence of the p38 mitogen-activated protein kinase (p38MAPK) inhibitor, SB203880 (10 μM; Sigma-Aldrich, Milan, Italy) and processed for messenger RNA (mRNA) extraction, as outlined above. In some experiments, liver nonparenchymal cells were separated on a Ficoll density gradient, stained with fluorochrome-conjugated Abs for CD45, F4/80, and IL-10 (eBiosciences, San Diego CA), and analyzed with a FACScalibur (Becton Dickinson, Franklin Lakes, NJ) flow cytometer. Unspecific immunoglobulin binding was blocked by incubation with decomplemented mouse serum. AnxA1-producing cells were detected using a polyclonal anti-AnxA1 rabbit antiserum (Millipore, Temecula, CA) and phycoerythrin-conjugated anti-rabbit IgG (Sigma-Aldrich).

### Western Blotting

Liver fragments were homogenized in ice-cold lysis buffer, as previously reported,^13^ and protein extracts (100 µg) were electrophoresed on a 10% SDS-polyacrylamide gel. Nitrocellulose membranes probed with monoclonal Abs against mouse AnxA1 and galectin-3 were revealed with Western Lightning Chemiluminescence Reagent Plus (ECL; Perkin-Elmer, Boston, MA) using the VersaDoc 3000 quantitative imaging system and Quantity One software (Bio-Rad).

### Statistical Analysis

Statistical analyses were performed by SPSS statistical software (SPSS, Inc., Chicago IL, USA) using the one-way analysis of variance (ANOVA) test with Tukey's correction for multiple comparisons or Kruskal-Wallis' test for nonparametric values. Significance was taken at the 5% level. Normality distribution was preliminarily assessed by Kolmogorov-Smirnov's test.

## Results

### The Progression of NASH Is Associated With a Stimulation in Liver AnxA1

**Fig 1:**
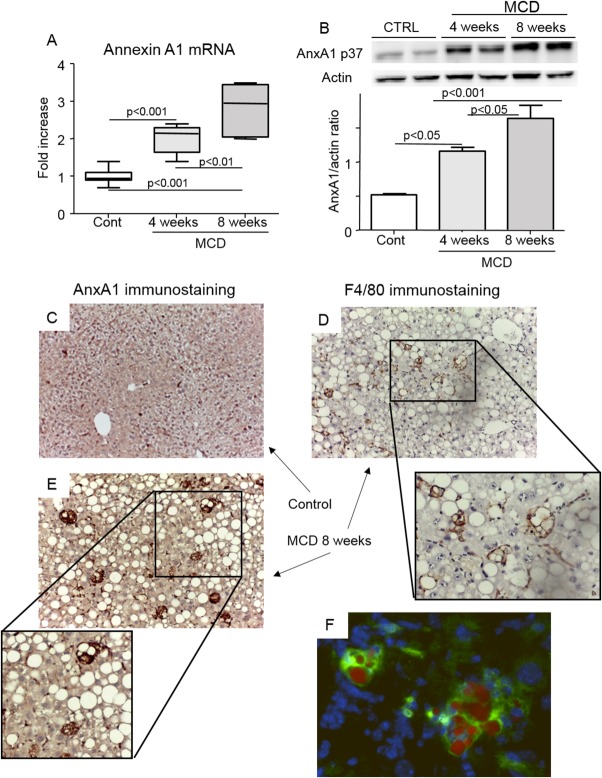
Hepatic AnxA1 expression in mice with NASH. Mice were fed an MCD diet over an 8-week period. (A and B) AnxA1 mRNA and protein levels, as measured by RT-PCR and western blotting analyses, respectively, in liver extracts of mice receiving control or MCD diet. Hepatic mRNA data are expressed as fold increase over control values after normalization to the β-actin gene. Data are from 8-12 animals per group; boxes include the values within 25th and 75th percentile, whereas horizontal bars represent the medians. The extremities of the vertical bars (10th-90th percentile) comprise 80% of the values. Statistical differences were assessed by one-way ANOVA test with Tukey's correction for multiple comparisons. (C and D) Localization of AnxA1 expression by IHC in liver of MCD-fed animals (magnification, 400×). (E) Detection of macrophages positive for F4/80 immunostaining (magnification, 400×). (F) Colocalization of AnxA1 in macrophages containing lipid vacuoles was evidenced by double staining of frozen liver sections with the lipid dye, Oil Red O (red), and anti-AnxA1 Ab (green immunofluorescence; magnification, 400×). Cell nuclei were courterstained with 4′,6-diamidino-2-phenylindole. Images are representative of 3-4 distinct samples.

AnxA1 was expressed at low extent in livers of naïve mice, whereas both mRNA and protein content increased in a time-dependent manner in livers of animals with NASH induced by feeding the MCD diet (Fig. 1). AnxA1 expression in NASH livers was selectively localized in enlarged vacuolized mononucleated cells that were positive for the macrophage marker, F4/80 (Fig. 1). Double staining of frozen liver sections with an anti-AnxA1 Ab and the lipid dye Oil Red O confirmed that AnxA1-positive macrophages contained lipid droplets (Fig. 1), likely derived from the scavenging of dying fat-laden hepatocytes. Histological analysis indicated that AnxA1-producing cells were more frequent in livers of animals with advanced NASH (8 weeks on the MCD diet) than in those with less-severe steatohepatitis (4 weeks of treatments; 4.7 ± 0.5 vs. 2.8 ± 0.8 cells/high-magnification field [hmf]; *P* = 0.03). Animals with advanced NASH also showed an increase in hepatic mRNA content of the AnxA1 receptor, Fpr2/3, and presence of Fpr2/3-expressing cells in liver sections (Supporting Fig. 1). Although AnxA1 up-regulation paralleled the severity of NASH, an increased expression of AnxA1 was already evident along with early signs of inflammation in livers with only steatosis induced by feeding mice for 12 weeks with an HFD that caused obesity and IR (Supporting Fig. 2).

**Fig 2:**
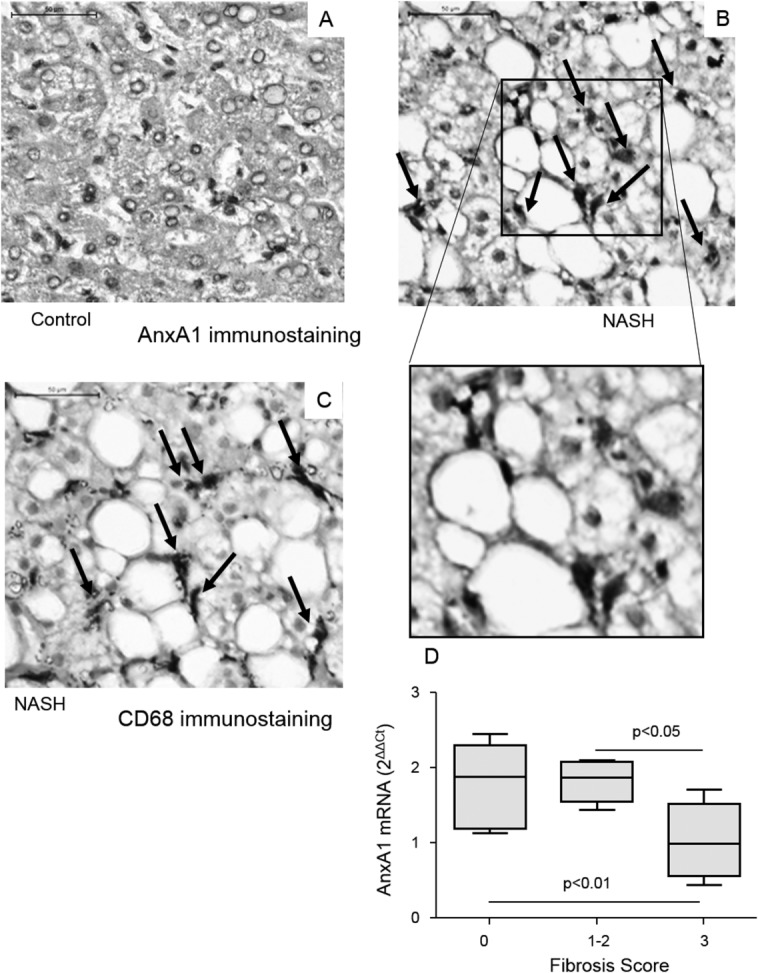
AnxA1 expression in human livers with or without NASH. AnxA1 detection by IHC in liver specimens from control individuals (A) and NASH patients (B). CD68-positive macrophages (from the same NASH patient; C) (magnification, 400×). Control liver samples refer to surgical resections for hepatic metastasis of colon carcinoma. (D) AnxA1 mRNA was measured in liver biopsies of 28 NASH patients using RT-PCR and normalized to that of β-actin. Boxes include the values within 25th and 75th percentile, whereas horizontal bars represent the medians. The extremities of the vertical bars (10th-90th percentile) comprise 80% of the values. The extent of liver fibrosis was scored according to Kleiner et al.^12^

In line with the results obtained in rodents, liver biopsy sections from subjects with NASH showed an increased prevalence of CD68^+^ macrophages producing AnxA1 (Fig. 2). A further evaluation of AnxA1 mRNA levels in 28 liver biopsies from NAFLD/NASH patients evidenced that AnxA1 expression was not related to the degree of IR or severity of liver injury (not shown), but inversely correlated with extension of fibrosis (r = −0.59; *P* < 0.003). In particular, AnxA1 mRNA was significantly lower in subjects with bridging fibrosis, as compared to those with mild/moderate pericentral or -portal fibrosis or without fibrosis (Fig. 2).

### AnxA1 Deficiency Stimulates Hepatic Inflammation in Advanced NASH

**Fig 3:**
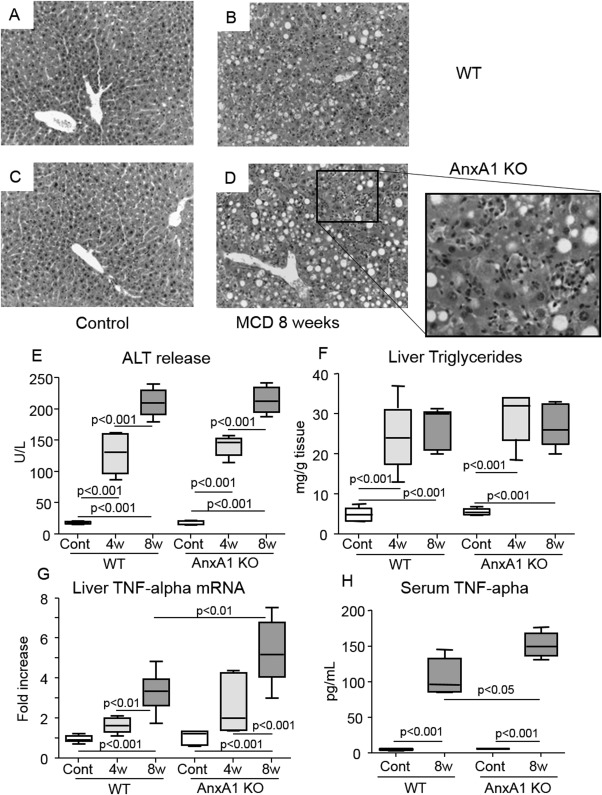
AnxA1 deficiency promotes steatohepatitis in mice with NASH. WT and AnxA1 KO C57BL/6 mice were fed the MCD diet up to 8 weeks. (A-D) Liver histology was evaluated in H&E-stained sections from control or MCD-fed animals (magnification, 200×). (E-G) NASH severity was assessed by circulating ALT release, hepatic TG content and liver TNF-α mRNA levels, measured by RT-PCR and expressed as fold increase over control values after normalization to the β-actin gene. (H) Circulating TNF-α levels were determined by ELISA. Values refer to 6-8 animals per group; boxes include the values within 25th and 75th percentile, whereas horizontal bars represent the medians. The extremities of the vertical bars (10th-90th percentile) comprise 80% of the values. Statistical differences were assessed by one-way ANOVA test with Tukey's correction for multiple comparisons.

**Fig 4:**
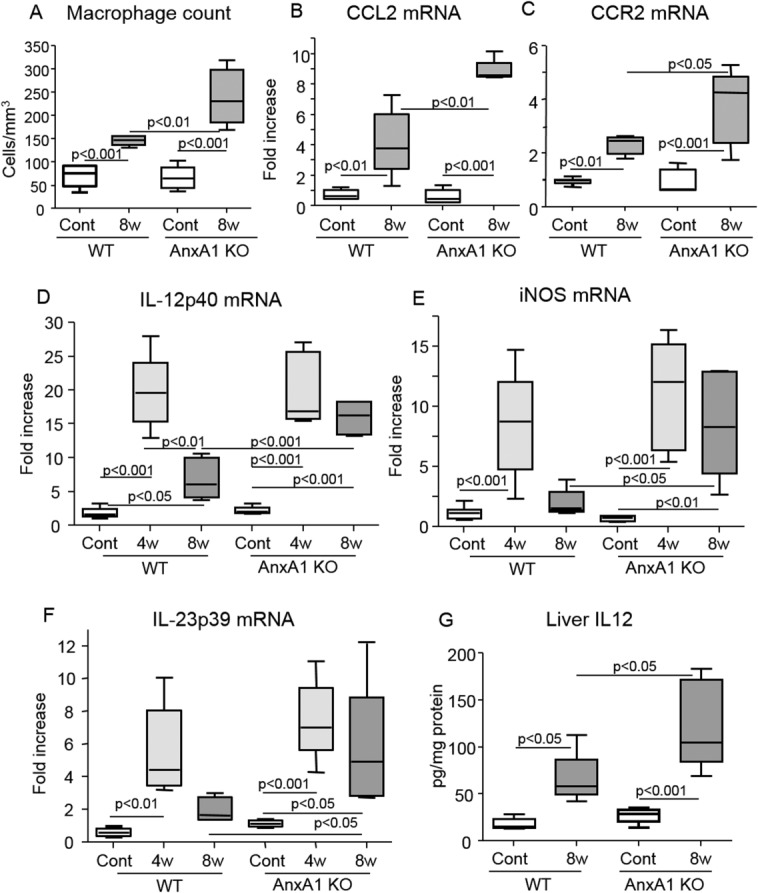
AnxA1 deficiency promotes liver macrophage recruitment and activation. WT and AnxA1 KO C57BL/6 mice were fed the MCD diet up to 8 weeks. (A) Macrophage counts after immunostaining with anti-F4/80 Ab. (B-F) Liver mRNA levels for CCL2, CCR2, and the macrophage M1 activation markers, IL-12p40, IL23p19, and iNOS, as measured by RT-PCR. Data are expressed as fold increase over control values after normalization to the β-actin gene. (G) Liver IL-12 protein content as determined in the same animals. In all cases, values refer to 8-12 animals per group and boxes include the values within 25th and 75th percentile, whereas horizontal bars represent the medians. The extremities of the vertical bars (10th-90th percentile) comprise 80% of the values. Statistical differences were assessed by one-way ANOVA test with Tukey's correction for multiple comparisons.

To further make insights on the role of AnxA1 in the evolution of NASH, AnxA1 KO mice were administered the MCD diet. Livers of AnxA1 KO mice on a control diet displayed normal histological appearance apart from the sporadic presence of monocyte infiltration (Fig. 3). Upon feeding the MCD diet for either 4 and 8 weeks, liver TGs, transaminase release, and the prevalence of apoptotic cells (4.2 ± 1.7 vs. 3.4 ± 0.6; *P* = 0.33) and necrotic foci (4.5 ± 1.4 vs. 3.8 ± 1.9; *P* = 0.43) in AnxA1 KO and WT mice were not statistically different (Fig. 3). However, already after 4 weeks on MCD diet, semiquantitative scores of lobular inflammation were higher in AnxA1 KO mice (2.2 ± 0.4 vs. 1.4 ± 0.5; n = 12; *P* = 0.01) and remained elevated by extending the treatment to 8 weeks (2.4 ± 0.6 vs. 1.6 ± 0.5; n = 12; *P* = 0.03). At this time point, NASH in AnxA1 KO mice was characterized by diffuse inflammatory foci containing mononucleated cells and by marked up-regulation in liver and circulating levels of TNF-α (Fig. 3). Accordingly, 8-week MCD-fed AnxA1 KO mice had higher mRNA for the chemokine, CCL2, and its receptor, CCR2, together with an elevated number of hepatic macrophages, when compared to paired WT animals (Fig. 4).

### Characterization of AnxA1 Effect on Hepatic Macrophages Functions

To further characterize the molecular and cellular events modulated by AnxA1, hepatic macrophage functions were investigated. In WT mice, NASH progression is characterized by a biphasic change in liver expression of macrophage M1 activation markers, including iNOS, IL-12p40, and IL-23p19. These were elevated after 4 weeks on the MCD diet and declined thereafter (Fig. 4). In these animals, individual AnxA1 mRNAs inversely correlated with those of iNOS (r = −0.62; *P* = 0.01), IL-12p40 (r = −0.48; *P* = 0.03), and IL-23p19 (r = −0.62; *P* = 0.03), pointing to the possible contribution of endogenous AnxA1 in down-regulating macrophage M1 responses during disease progression. Supporting this view, AnxA1 KO mice receiving the MCD diet for 8 weeks did not show any decline in iNOS, IL-12p40, and IL-23p19 liver mRNAs: These markers were instead approximately 2-3 fold higher than those measured in WT animals (Fig. 4). In the same vein, hepatic IL-12 protein content was significantly enhanced in AnxA1-KO mice (Fig. 4).

**Fig 5:**
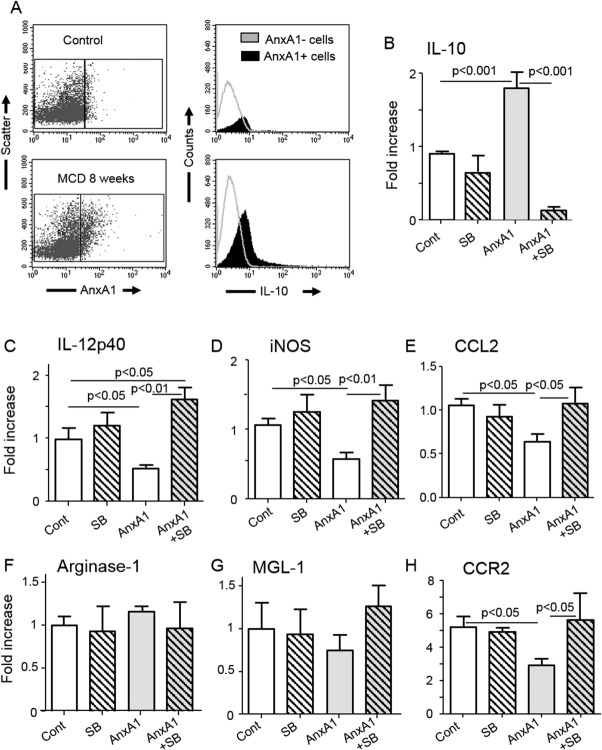
AnxA1 regulates macrophage functions through stimulation of IL-10 production. Hepatic macrophages were isolated from livers of either control or 8-week MCD-fed mice. (A) F4/80-positive cells were analyzed by FCM for the coexpression of AnxA1 and IL-10 or MCD-fed mice. (B) Macrophages isolated from livers of MCD-fed mice were incubated *in vitro* with or without recombinant AnxA1 (100 nM) and the p38MAPK inhibitor, SB203880 (SB; 10 μM), and subsequently analyzed for expression of IL-10 mRNA (B). (C and D) RT-PCR analyses for other markers, including M1 (iNOS and IL-12p40) and (F and G) M2 activation markers (arginase-1 and MGL-1/CD301), as well as (E and H) CCL2 or CCR2. Values refer to four to five different cell preparations and are presented as mean ± standard deviation. Statistical differences were assessed by one-way ANOVA test with Tukey's correction for multiple comparisons.

Recent reports have linked the proresolving properties of AnxA1 with a stimulation of IL-10 production through a p38MAPK signaling path, together with induction of M2 polarization.^16^,^17^ FCM analysis of macrophages isolated from livers of WT mice fed for 4 weeks with the MCD diet showed that AnxA1-expressing F4/80^+^ cells had a higher IL-10 content than those with AnxA1 negatives (Fig. 5). Furthermore, incubation with AnxA1 of macrophages isolated from NASH livers halved the expression of iNOS and IL-12p40 without affecting that of the M2 markers, arginase-1 and MGL-1/CD301 (Fig. 5). In AnxA1-treated macrophages, suppression of M1 activation was associated with a 2-fold increase in IL-10 mRNA (Fig. 5). Moreover, addition of the p38MAPK inhibitor, SB203880, reverted both AnxA1-induced IL-10 stimulation and down-modulation of iNOS and IL-12p40 (Fig. 5). Interestingly, macrophage incubation with AnxA1 also significantly lowered CCL2 and CCR2 mRNA levels in a p38MAPK-dependent manner (Fig. 5), suggesting the potential capacity of AnxA1 to influence hepatic monocytic recruitment through CCL2 and CCR2 signalling.

### AnxA1 Deficiency Stimulates the Fibrogenic Evolution of NASH

**Fig 6:**
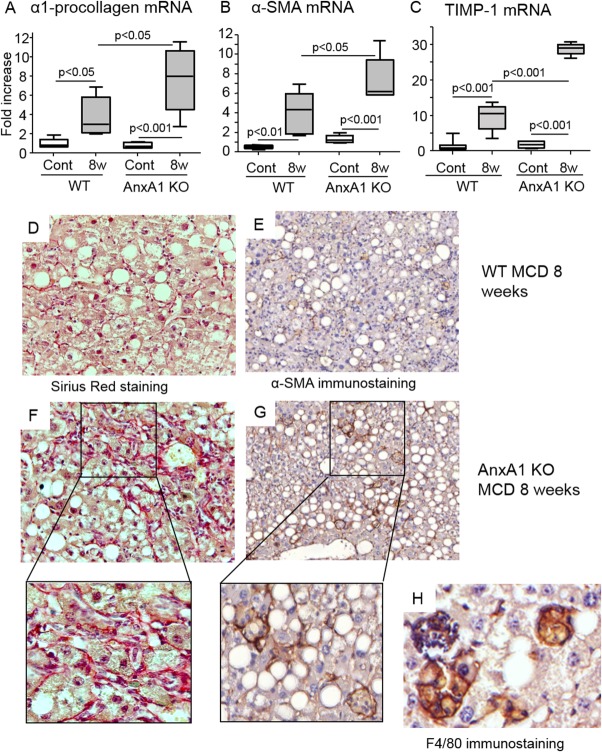
AnxA1 deficiency promotes hepatic fibrosis in mice with NASH. WT and AnxA1 KO C57BL/6 mice were fed the MCD diet for 8 weeks. (A-C) Liver mRNA levels for pro-collagen-1α, α-SMA, and TIMP-1, as measured by RT-PCR, and are expressed as fold increase over control values after normalization to the β-actin gene. Values refer to 6-8 animals per group and boxes include the values within 25th and 75th percentile, whereas horizontal bars represent the medians. The extremities of the vertical bars (10th-90th percentile) comprise 80% of the values. Statistical differences were assessed by one-way ANOVA test with Tukey's correction for multiple comparisons. (D and F) Collagen deposition as detected by Sirius Red staining in representative liver sections from 8-week MCD diet in WT and AnxA1 KO mice. (E and G) Activated HSCs expressing α-SMA (magnification, 400× and 200×). Enlargement shows α-SMA-positive HSCs surrounded by collagen fibers forming cell foci with mononucleated cells. (H) These latter were stained by the macrophage marker, F4/80 (magnification, 600×).

The data obtained in the patients with NAFLD/NASH indicate an inverse association between liver expression of AnxA1 and the extension of hepatic fibrosis. By investigating the effects of AnxA1 deficiency on the fibrogenic evolution of experimental NASH, we observed that the fibrosis markers, pro-collagen-1α, α-SMA, and TIMP-1, were higher in MCD-fed AnxA1 KO mice than in WT animals (Fig. 6). Hepatic collagen deposition, as evidenced by Sirius Red staining, and α-SMA-positive activated HSCs (4.3 ± 0.9 vs. 20.3 ± 3.8 cells/hmf; *P* < 0.005) were also more evident in AnxA1 KO mice with NASH. These latter α-SMA-positive HSCs were often surrounded by cell clusters containing F4/80-positive mononucleated cells (Fig. 6). Stimulation of liver fibrosis in AnxA1-KO animals was unrelated to regulation of hepatic TGF-β1 or of MMP-9 and MMP-13 (Supporting Fig. 3), suggesting that additional factors may contribute to the profibrogenic evolution of NASH.

**Fig 7:**
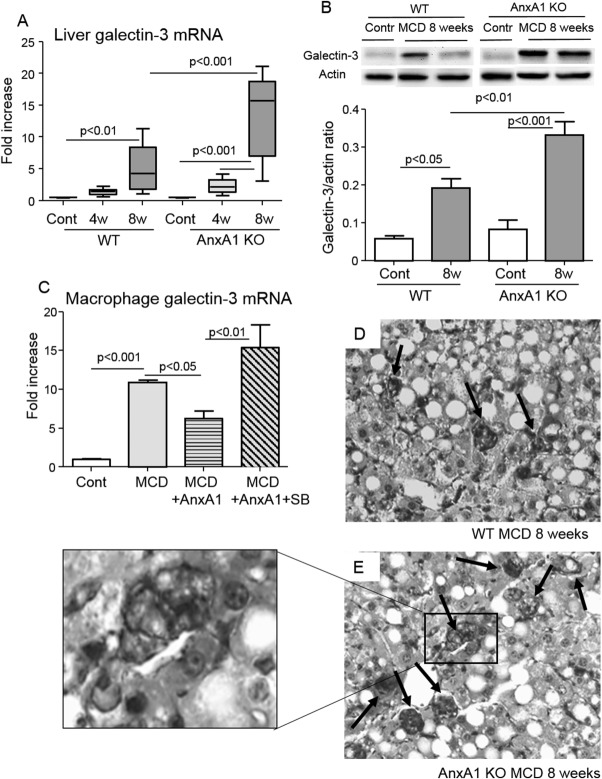
AnxA1 regulates galectin-3 production in livers of mice with NASH. WT and AnxA1 KO C57BL/6 mice were fed the MCD diet for 8 weeks. (A and B) Galectin-3 mRNA (boxes and whiskers) and protein levels (mean ± standard deviation) were measured by RT-PCR and western blotting, respectively. Data are from 6-8 mice per group and statistical differences were assessed by one-way ANOVA test with Tukey's correction for multiple comparisons. (C) Macrophages isolated from livers of control (Cont) or 4-week MCD-fed mice were incubated with or without recombinant AnxA1 (100 nM) and the p38MAPK inhibitor, SB203880 (SB; 10 μM), for 16 hours before quantification of galectin-3 mRNA. (D and E) Liver galectin-3 expression by IHC in MCD-fed animals (magnification, 400×). Enlargement shows cell foci containing galectin-3 macrophages and HSCs.

The beta-galactoside-binding lectin, galectin-3, can direct myofibroblast activation in fibrotic livers and has been associated with the pathogenesis of NASH.^18^,^19^ In our experiments, liver galectin-3 expression was up-regulated in WT mice with advanced NASH and even more in AnxA1 KO mice which, at the 8-week time point had a hepatic content of galectin-3 2-fold higher than WT mice (Fig. 7). In line with these *ex vivo* data, *in vitro* experiments showed that AnxA1-mediated signals effectively down-modulated galectin-3 expression in macrophages isolated from NASH livers (Fig. 7). Conversely, addition of AnxA1 to the same macrophage preparations did not affect TGF-β1 mRNA (not shown). At immunohistochemistry (IHC), galectin-3 production was particularly evident in the macrophage foci mentioned above (Fig. 7). These galectin-3-positive foci were more frequent in AnxA1 KO livers (2.8 ± 0.8 vs. 4.6 ± 0.5 cell foci/hmf; *P* < 0.03). Moreover, in these latter, the presence of HSCs along with the macrophages is associated with abundant collagen fibers surrounding the foci (Fig. 6).

## Discussion

Growing evidence points to the importance of AnxA1 in the modulation of anti-inflammatory and proresolving responses in rodent models of acute inflammation.^6^,^7^ We now identify novel modulatory functions of AnxA1 in livers of mice with chronic steatohepatitis induced by feeding with an MCD diet. Coupled to the translational data with human NASH, and a series of mechanistic readouts in human and mouse macrophage, we unveil an AnxA1-centerd pathway engaged by the host for liver protection.

In experimental models of NAFLD/NASH, up-regulation of AnxA1 is already evident in fatty livers and further increases with disease progression specifically involving macrophages containing intracytoplasmatic lipid droplets. The origin of these macrophages has not been characterized in detail, though our preliminary data indicate that these cells express, to a low extent, the monocyte marker, Ly6C,^20^ suggesting that they might derive from inflammatory macrophages that had undergone phenotypic changes after scavenging dying fat-laden hepatocytes. Interestingly, macrophages are also the predominant source of AnxA1 in adipose tissue of obese mice,^12^ suggesting that, during MetS, AnxA1 up-regulation might be a common response in macrophages, irrespective of their tissue location. In this study, the increase in liver AnxA1 was observed in both an experimental model of NAFLD based on mice feeding with an HFD as well as in NASH induced by the MCD diet. We are aware that the MCD model does not reproduce some of the key features of human diseases, such as obesity and IR. However, it was preferred for characterizing the role of AnxA1 in NASH evolution because it causes extensive steatohepatitis rapidly progressing to fibrosis.^21^

In line with the homeostatic properties of AnxA1, we observed that AnxA1 deficiency promotes lobular inflammation in MCD-fed mice, particularly in animals with more advanced NASH, as observed after 8 weeks of treatment. The mechanisms behind the inflammatory phenotype appear related to the direct action of AnxA1 on the macrophages. Indeed, in livers of WT animals with advanced NASH, higher AnxA1 expression is associated with a down-modulation of the macrophage M1 phenotype. Such an effect is absent in AnxA1 KO mice that display instead elevated expression of iNOS, IL-12p40, and IL-23p19 gene products. Moreover, the *in vitro* addition of recombinant AnxA1 reduced M1 marker expression in macrophages isolated from NASH livers by more than 50%. Altogether, these results suggest that macrophage-derived AnxA1 represents an autocrine/paracrine loop that suppresses, at least in part, proinflammatory M1 responses. This new notion is consistent with data indicating a central role for AnxA1 in the down-modulation of TNF-α and IL-6 production by macrophages exposed to glucocorticoids.^6^,^22^

The homeostatic functions of AnxA1 are mediated by FPR2/ALX, a G-protein-coupled receptor that is shared with other proresolving lipid mediators, including lipoxin A_4_ and resolvin D1,^23^,^24^ as well as with the proinflammatory protein, serum amyloid A, and cathelicidin LL-37.^25^,^26^ We observed that *in vivo* macrophage responses to endogenous AnxA1 become appreciable when FPR2/ALX is overexpressed in advaced NASH. Recently, Cooray et al. unveiled an AnxA1-specific FRP2/ALX proresolving signal pathway involving p38MAPK, MAPKAPK1/2, and heat shock protein 27, leading to generation of IL-10.^17^ In our experiments with isolated macrophages from NASH livers, down-modulation of iNOS and IL-12p40 is associated with a 2-fold rise in IL-10 expression; importantly, pharmacological inhibition of p38MAPK affects, in opposite ways, AnxA1-induced IL-10 stimulation and suppression of M1 polarization. The importance of IL-10 in mediating AnxA1 action is consistent with the observation that an *N*-terminal AnxA1-derived peptide (Ac1-25) fails to exert anti-inflammatory activity in IL-10-deficient mice.^27^ Collectively, and complementing a recent study,^17^ these data indicate that AnxA1 can down-regulate macrophage M1-responses through an FPR2/ALX dimerization signal centerd on p38 and ultimately leading to IL-10 generation. These new data provide pathophysiological relevance to this novel network of resolution, formed by AnxA1/FPR2 and IL-10, and operative in NASH livers to limit and delay disease progression.

In addition to modulation of proinflammatory activity of macrophages, AnxA1 might also regulate liver recruitment of monocytes by controlling the expression of the CCL2/CCR2 pair. Indeed, CCR2 is one of the genes more extensively down-regulated in human monocytes exposed to Ac1-25 AnxA1-derived peptide.^28^ However, differently from that reported by Lange et al.,^28^ in our hands, the p38 inhibitor, SB203880, effectively reverted the effect of AnxA1 on CCR2 regulation in mice macrophages. Thus far, AnxA1 has been implicated in reducing granulocyte-tissue infiltration by blocking neutrophil-endothelial interactions and accelerating neutrophil apoptosis.^6^,^7^ Neutrophil infiltration is not relevant in the pathogenesis of NASH, whereas macrophages and lymphocytes are the predominant inflammatory cells.^29^ Thus, our data point to the possibility that, during chronic inflammation, AnxA1 might also control the recruitment of monocytes. The capacity of AnxA1 to interfere with the CCL2/CCR2 axis might be particularly relevant in relation to the evolution of steatohepatitis, because CCR2 deficiency and CCR2 antagonism ameliorate liver injury and fibrosis in experimental NASH.^30^,^31^ Possibly unrelated to the IL-10 effect discussed above, AnxA1-mediated CCR2 down-regulation provides further mechanistic support to the “protective” role played by this mediator.

NASH is increasingly recognized as an important cause for liver fibrosis, and approximately 15% of NASH patients progress to advanced fibrosis/cirrhosis.^1^ However, the factors responsible for the large interindividual variability in development of fibrosis are still poorly characterized. Here, we report that (1) AnxA1-expressing macrophages are evident in liver biopsies of patients suffering from NAFLD/NASH and (2) AnxA1 mRNA levels in these patients inversely correlate with the severity of fibrosis. In line with these clinical data, experimental NASH in AnxA1-KO mice is characterized by increased liver fibrosis, suggesting that AnxA1 can prevent the fibrogenic evolution of NASH. Besides the immune-mediated events discussed above, this effect might involve modulation of galectin-3 expression. Galectin-3 is a member of the galectin family, a group of lectins that participates in the regulation of cell adhesion, proliferation and survival, as well as in the modulation of tissue inflammation and fibrosis.^32^ In acutely injured livers, galectin-3 is mainly produced by macrophages and sustains M1 activation in models of acetaminophen- and concanavalin-A-induced hepatitis.^33^,^34^ Conversely, in rodent and human livers with fibrosis, galectin-3 is also expressed by activated α-SMA-positive HSCs and regulates their profibrogenic activity.^19^,^35^ Recent studies have implicated galectin-3 in the pathogenesis of NASH, although with controversial results, because galectin-3-deficient mice show either protection^18^ or increased disease severity^36^,^37^ in relation to an impaired glucose metabolism and an increased susceptibility to obesity and systemic inflammation.^38^,^39^ In our hands, an increase in liver galectin-3 characterizes advanced NASH in MCD-fed mice and galectin-3 appears susceptible to modulation by AnxA1. Moreover, the worsening of fibrosis quantified in MCD-fed AnxA1 KO mice associates with a further up-regulation in galectin-3 levels. In these latter, hepatic fibrosis is characterized by the diffuse presence of cell clusters composed of galectin-3-expressing macrophages and activated HSCs, whereas liver collagen deposition is particularly evident around these clusters, supporting a specific role of galectin-3 in stimulating profibrogenetic cell-to-cell interactions. Consistently, galectin-3 secretion by macrophages has been implicated in the promotion of renal and vascular fibrosis,^40^,^41^ whereas genetic deletion or inhibition of galectin-3 attenuates HSC activation and hepatic collagen deposition in CCl_4_^−^ or tioacetamide-treated mice^19^,^42^ and ameliorated inflammation and fibrosis in experimental NASH.^43^

Altogether, these results afford a novel functional role for AnxA1 in NASH progression, a property mediated through a control of hepatic inflammation and fibrogenesis as well as uneven modulation of galectin-3 and IL-10, leading to a reduced macrophage M1 response. It is plausible that strategies aiming at increasing hepatic AnxA1 expression, or the development of AnxA1 analogs,^44^ might have a potential for therapeutic control on NASH evolution.

## Abbeviations

Abs: antibodies
ALT: alanine aminotransferase
ANOVA: analysis of variance
AnxA1: Annexin A1
CCL2: chemokine (C-C motif) ligand 2
CCR2: C-C chemokine receptor type 2
cDNA: complementary DNA
ELISA: enzyme-linked immunosorbent assay
FCM: flow cytometry
FPR2/ALX;: formyl peptide receptor 2/lipoxin A4 receptor
H&E: hematoxilin and eosin
HFD: high-fat diet
hmf: high-magnification field
HSCs: hepatic stellate cells
IgG: immunoglobulin G
IHC: immunohistochemistry
IL: interleukin
iNOS: inducible nitric oxide synthase
IR: insulin resistance
KO: knockout
MCD: methionine-choline deficient
MetS: metabolic syndrome
MGL-1: macrophage galactose *N*-acetyl-galactosamine-specific lectin-1
MMP: matrix metalloproteinase
mRNA: messenger RNA
NAFLD: nonalcoholic fatty liver disease
NASH: nonalcoholic steatohepatitis
NO: nitric oxide
p38MAPK: p38 mitogen-activated protein kinase
RT-PCR: real-time polymerase chain reaction
α-SMA: alpha-smooth muscle actin
TGs: triglycerides
TGF-β1: transforming growth factor beta 1
TIMP-1: tissue inhibitor of metalloproteinase-1
TNF-α: tumor necrosis factor alpha
WT: wild type
