# Supplementary material for: Endogenous annexin A1 is a novel protective determinant in nonalcoholic steatohepatitis in mice
Source: Hepatology. 2014 May 12;60(2):531–44. doi: 10.1002/hep.27141 (PMC4258084; doi:10.1002/hep.27141)
Supplement: Supplementary file 1 [file hep0060-0531-SD1.pdf]

Locatelli et al. ENDOGENOUS ANNEXIN A1 IS A NOVEL PROTECTIVE DETERMINANT IN NONALCOHOLIC STEATOHEPATITIS (NASH)

Supplementary Material

Material and Methods

Animal and Experimental protocol. In some experiments 8 week old C57BL/6 male mice were feed "ad libitum" for 12 weeks with a liquid high fat diet according to the standard Liber-De Carli formulation (Hall et al. 2001). The diet contained carbohydrates, as maltose dextrins (48% of total Kcal, proteins, as casein (17% of total Kcal) and fat, as corn oil (26% of total Kcal) and was supplied by Laboratorio Dottori Piccioni (Gessate, Italy). This dietary protocol induced obesity, insulin resistance and fatty liver without appreciable hepatic injury as measured by transaminase release (Supplementary Fig. 2). Insulin resistance was evaluated by monitoring blood glucose up to 120 min after IP injection of glucose (1.5 g/Kg bwt) or insulin (0.75 U/Kg bwt).

Hall P, Lieber CS, De Carli LM, French SW, Lindros KO, Javerlanen H, Bode C, Parlesak A, Bode JC. Models of alcoholic liver disease in rodents: a critical evaluation. Alcohol Clin Exp Res 2001;25:245S-261S.

Supplementary Table 1

Clinical and biochemical characterization of NAFLD/NASH patients investigated**.**

	Demographic Data
Patients Number (Male/Female)	28 (22/6)
Age (Years)	44.3 (29-66)
BMI	26.6 (17-31)
	Biochemical Data
HOMA-IR (n.v. <3)	5.18 (0.4-13.7)
AST (U/L– n.v. 5–40)	43.6 (15-198)
ALT (U/L n.v. 5–40)	77.0 (13-323)
γ-GT (U/L n.v. 5–45)	118.5 (12-792)
Fasting Glucose (mg/dL n.v. <100)	100.6 (78-245)
	Histological Data
Steatosis score	1.5 (1-3)
Inflammation score	0.7 (0-2)
Ballooning score	1 (0-2)
Fibrosis score	1.4 (0-3)
NAS score	3.2 (1-5)

The values are expressed as median and inter-quartile range (IQR). For histological scores the range of variability is included.

BMI, body mass index; AST, alanine aminotransferase; ALT, aspartate aminotransferase; γ-GT, gamma-glutamyl transpeptidase; HOMA-IR, homeostatic model assessment-insulin resistance; ISI, insulin sensitivity index; n.v., normal value

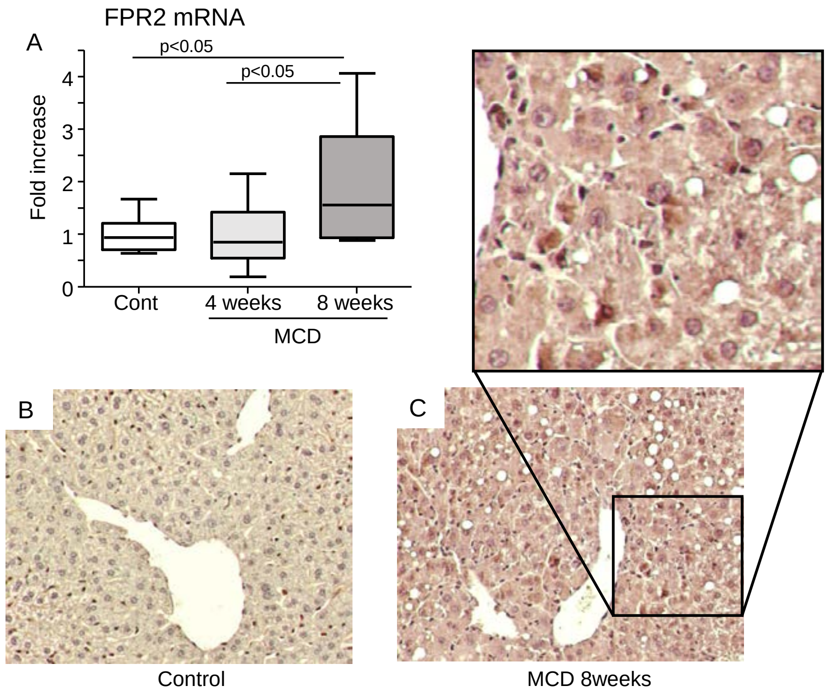

Supplementary Figure 1

Expression of AnxA1 receptor Fpr2 in the livers of mice with NASH induced by feeding a methionine-choline deficient (MCD) diet.

Wild type C57BL/6 mice received the MCD diet up to 8 weeks. (A) Fpr2 mRNA was measured by RT-PCR and expressed as fold increase over control values after normalization to the β-actin gene (panel A). The values refer to 6-8 animals per group and the boxes include the values within 25th and 75th percentile, while the horizontal bars represent the medians. The extremities of the vertical bars (10th-90th percentile) comprise the eighty percent of the values. Statistical differences were assessed by one-way ANOVA test with Tukey's correction for multiple comparisons. (B,C) Fpr2 liver localization by immunohistochemistry in control or MCD-fed mice.

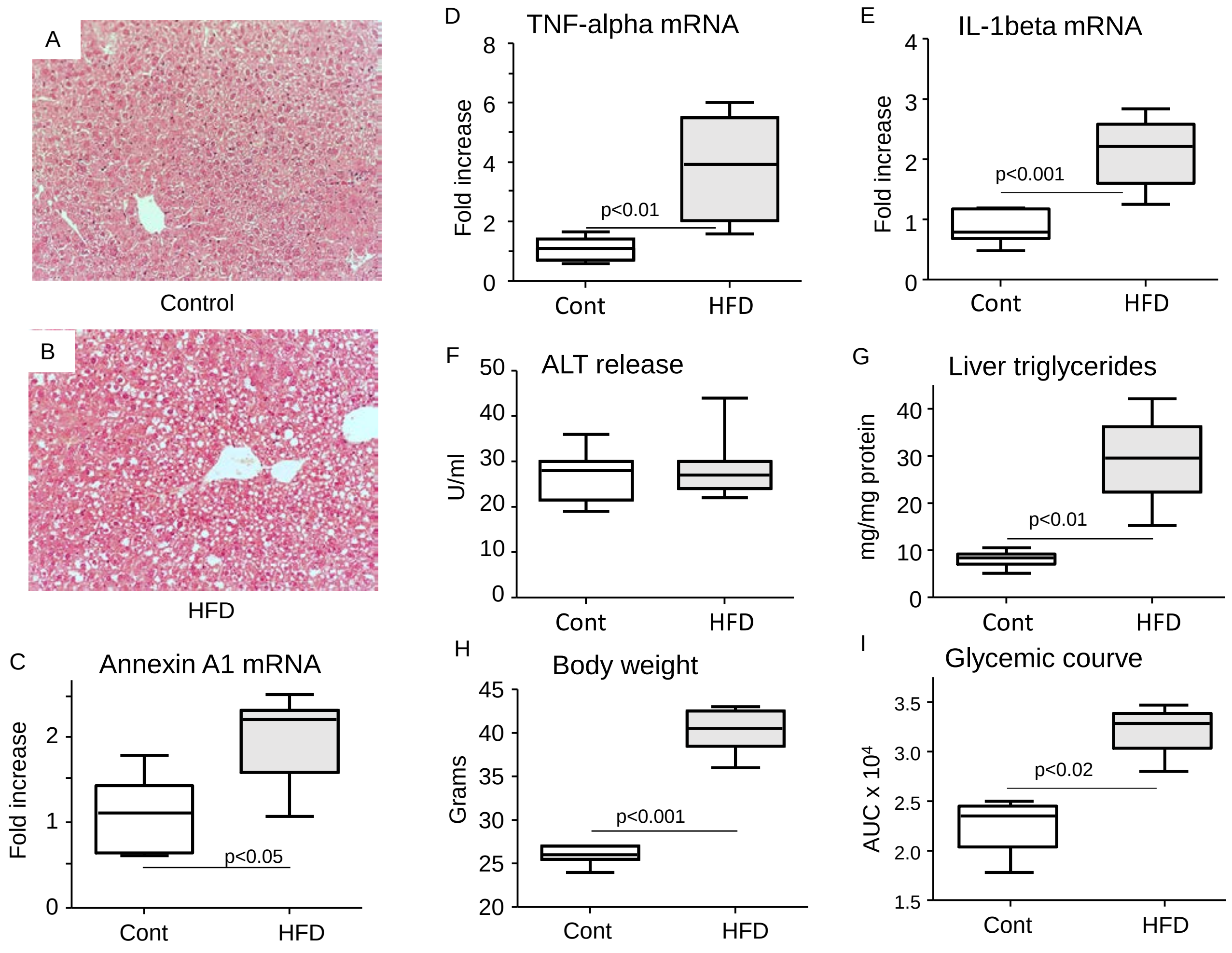

Supplementary Figure 2

Expression of AnxA1 in the livers of mice with liver steatosis induced by feeding a high fat diet (HFD).

Wild type C57BL/6 mice received the HFD diet for 12 weeks. (A,B) Liver histology was evaluated in hematoxilin/eosin stained sections from control or HFD-fed animals (magnification 200x). (C-E) AnxA1, TNF-α and IL-1-β mRNAs were measured by RT-PCR and expressed as fold increase over control values after normalization to the β-actin gene. (F,G) Alanine aminotransferase (ALT) release, hepatic triglyceride content by enzymatic methods. (H,I) Body weight and glycemic curve were monitored at the end of the treatment. The values refer to 5-6 animals per group and the boxes include the values within 25th and 75th percentile, while the horizontal bars represent the medians. The extremities of the vertical bars (10th-90th percentile) comprise the eighty percent of the values.

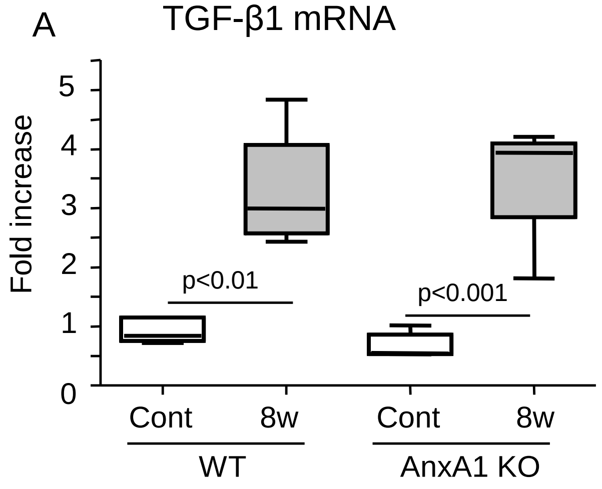

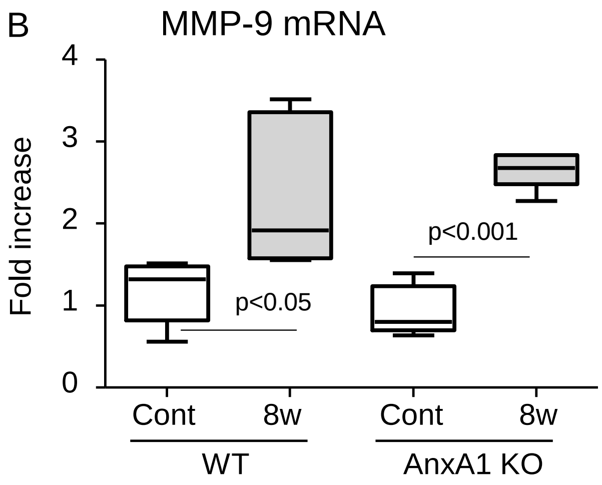

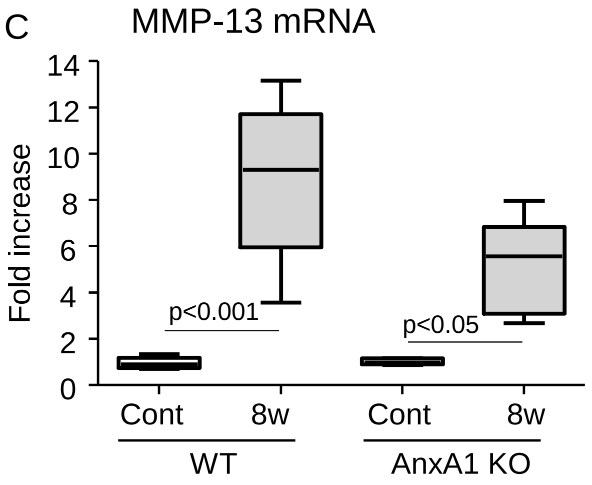

Supplementary Figure 3

Expression of TGF-β1 and metalloproteases (MMP) 9 and 13 in the livers of WT or AnxA1 KO mice with NASH induced by feeding a methionine-choline deficient (MCD) diet.

Hepatic mRNA levels were measured by RT-PCR and expressed as fold increase over control values after normalization to the β-actin gene. The values refer to 6-8 animals per group; boxes include the values within 25th and 75th percentile, while the horizontal bars represent the medians. The extremities of the vertical bars (10th-90th percentile) comprise the eighty percent of the values. Statistical differences were assessed by one-way ANOVA test with Tukey's correction for multiple comparisons.
